# Ibuprofen regulates the expression and function of membrane-associated serine proteases prostasin and matriptase

**DOI:** 10.1186/s12885-015-2039-6

**Published:** 2015-12-29

**Authors:** Andreas C. Chai, Andrew L. Robinson, Karl X. Chai, Li-Mei Chen

**Affiliations:** Burnett School of Biomedical Sciences, University of Central Florida College of Medicine, 4000 Central Florida Boulevard, Building 20, Room 323, Orlando, FL 32816-2364 USA

**Keywords:** Ibuprofen, Prostasin, Matriptase, Cyclooxygenase, Tight junction, Cancer

## Abstract

**Background:**

The glycosylphosphatidylinositol-anchored extracellular membrane serine protease prostasin is expressed in normal bladder urothelial cells. Bladder inflammation reduces prostasin expression and a loss of prostasin expression is associated with epithelial-mesenchymal transition (EMT) in human bladder transitional cell carcinomas. Non-steroidal anti-inflammatory drugs (NSAIDs) decrease the incidence of various cancers including bladder cancer, but the molecular mechanisms underlying the anticancer effect of NSAIDs are not fully understood.

**Methods:**

The normal human bladder urothelial cell line UROtsa, the normal human trophoblast cell line B6Tert-1, human bladder transitional cell carcinoma cell lines UM-UC-5 and UM-UC-9, and the human breast cancer cell line JIMT-1 were used for the study. Expression changes of the serine proteases prostasin and matriptase, and cyclooxygenases (COX-1 and COX-2) in these cells following ibuprofen treatments were analyzed by means of reverse-transcription/quantitative polymerase chain reaction (RT-qPCR) and immunoblotting. The functional role of the ibuprofen-regulated prostasin in epithelial tight junction formation and maintenance was assessed by measuring the transepithelial electrical resistance (TEER) and epithelial permeability in the B6Tert-1 cells. Prostasin’s effects on tight junctions were also evaluated in B6Tert-1 cells over-expressing a recombinant human prostasin, silenced for prostasin expression, or treated with a functionally-blocking prostasin antibody. Matriptase zymogen activation was examined in cells over-expressing prostasin.

**Results:**

Ibuprofen increased prostasin expression in the UROtsa and the B6Tert-1 cells. Cyclooxygenase-2 (COX-2) expression was up-regulated at both the mRNA and the protein levels in the UROtsa cells by ibuprofen in a dose-dependent manner, but was not a requisite for up-regulating prostasin expression. The ibuprofen-induced prostasin contributed to the formation and maintenance of the epithelial tight junctions in the B6Tert-1 cells. The matriptase zymogen was down-regulated in the UROtsa cells by ibuprofen possibly as a result of the increased prostasin expression because over-expressing prostasin leads to matriptase activation and zymogen down-regulation in the UROtsa, JIMT-1, and B6Tert-1 cells. The expression of prostasin and matriptase was differentially regulated by ibuprofen in the bladder cancer cells.

**Conclusions:**

Ibuprofen has been suggested for use in treating bladder cancer. Our results bring the epithelial extracellular membrane serine proteases prostasin and matriptase into the potential molecular mechanisms of the anticancer effect of NSAIDs.

## Background

Serine proteases have very diverse functions in biological and pathological processes, such as blood coagulation, complement activation, food digestion, blood pressure regulation, inflammation, and cancer [1]. Prostasin is a glycosylphosphatidylinositol (GPI)-anchored extracellular membrane serine protease with broad expression in all epithelial cells in many tissues and organs including the prostate, bladder, kidneys, colon, lungs, placenta, and skin [2]. Prostasin can also be detected in the urine and semen upon proteolytic shedding from the membrane. In the past 20 years since the discovery of prostasin [3–5], this protease has been shown to have important structural and/or functional roles in placental development, epithelial tight junction formation, epidermal/epithelial terminal differentiation, epithelial sodium channel activation, blood pressure regulation, and inflammation [2].

Prostasin has also been implicated for a role in many cancers including prostate, breast, ovarian, and bladder cancers. Prostasin expression is reduced in high-grade prostate cancers as well as in invasive human prostate and breast cancer cells [6–8] and bladder cancers [9]. But prostasin is over-expressed in the cancerous ovarian epithelial cells and stroma [10]. A loss of prostasin expression is associated with epithelial-mesenchymal transition (EMT) in human urothelial cancer cell lines and also correlates with the grades of bladder cancer [9]. On the other hand, re-expression of prostasin in cancer cells negative for prostasin could suppress tumor invasion and potentially metastasis [6, 7].

Transcription of the prostasin gene can be regulated by DNA methylation and histone acetylation [7, 9, 11], aldosterone [12], nerve growth factor (NGF, 11), transforming growth factor- β1 (TGF-β1, [13]), Slug [14], and sterol regulatory element-binding proteins (SREBPs) [15]. Further, in a lipopolysaccharide (LPS)-induced mouse bladder inflammation model, the prostasin gene expression was down-regulated and this down-regulation was associated with a marked increase in the expression of the inducible nitric oxide synthase (iNOS), cyclooxygenase-2 (COX-2), and some cytokines [16]. Over-expression of prostasin can attenuate LPS-induced iNOS up-regulation in the mouse bladder [16] and decrease the expression of iNOS and COX-2 genes in prostate cancer cells [17]; whereas silencing the prostasin gene expression in human prostate cells is associated with an induction of iNOS expression [18]. Prostasin can activate the type-II trans-membrane extracellular serine protease matriptase [19], while prostasin can also be activated reciprocally by matriptase [20]. The protease activity of prostasin can be regulated by reversible serine protease inhibitors such as the hepatocyte growth factor activator inhibitors, HAI-1 and HAI-2 [21, 22], or the irreversible serine protease inhibitor, protease nexin-1 (PN-1), [11].

Prostasin is normally localized at the apical side of terminally differentiated epithelial cells and is essential for epithelial tight junction functions [23]. Tight junctions are membranes joined together from adjacent cells on the apical end. The joint membranes create an impermeable barrier to seal the epithelium and separate the “inside” (interstitial space) from the “outside” (lumen or environment) of the body. The main structural proteins that constitute the epithelial tight junctions are the occludins [24] and the claudins [25]. Even though prostasin is required for the formation and functions of the tight junction, the exact molecular mechanisms of prostasin in this role are not clear, especially in regard to the epithelial tight junction proteins occludins and claudins. It is also not perfectly clear how prostasin’s expression and function are regulated in this role.

LPS can compromise epithelial tight junction and increase permeability via a mechanism dependent on the toll-like receptor 4 (TLR4) [26]. Prostasin expression is reduced by LPS [16] while prostasin can proteolytically cleave the TLR4 ectodomain (ECD) to down-modulate cellular signaling mediated by this receptor [27]. The attenuation of LPS-induced inflammatory mediator expression by forced prostasin expression [16] can potentially be attributed to prostasin’s regulatory role on the TLR4. Restoring prostasin expression in cells in the state of inflammation with a compromise of the tight junction barrier may then work toward restoring the barrier, in addition to taming the expression of the inflammatory mediators. In the clinical setting, prostasin expression may be manipulated by way of a pharmaceutical agent. The non-steroidal anti-inflammatory drugs (NSAIDs) decrease the incidence of various cancers including that of the colon, breast, lung and prostate, but the molecular mechanisms underlying the anticancer effect of NSAIDs are not fully understood [28]. The NSAID ibuprofen has been shown to reduce the survival of bladder cancer cells via the induction of the p75 neurotrophin receptor (p75^NTR^), a tumor and metastasis suppressor [29], and an inducer of prostasin expression [11]. We sought to investigate if prostasin can be regulated by ibuprofen in normal human bladder urothelial cells and in bladder cancer cells. We also tested if the ibuprofen-induced prostasin participates in the formation of epithelial cell tight junction using a normal human trophoblast cell line. Our study revealed that prostasin can be up-regulated by ibuprofen, and a properly regulated level of the prostasin protein in epithelial cells is critical for maintaining a healthy epithelial structure and function.

## Methods

The immortalized normal human urothelial cell line UROtsa was kindly provided by Dr. Donald A. Sens of the University of North Dakota, School of Medicine (Grand Forks, ND). The UM-UC-5 and UM-UC-9 human transitional cell carcinoma (TCC) cell lines were kindly provided by Dr. H. Barton Grossman (MD Anderson Cancer Center, Houston, TX). The hTERT-immortalized normal human trophoblast cell line B6Tert-1 was a gift of Dr. Yanling Wang (Institute of Zoology, State Key Laboratory of Reproductive Biology, Chinese Academy of Sciences, Beijing, China). The human breast cancer cell line JIMT-1 was purchased from the German Collection of Microorganisms and Cell Cultures (Braunschweig, Germany). The cell lines used in this study are publicly available and all information regarding the cell lines is also publicly available, and thus are not considered human subjects per guidelines of the National Institutes of Health (U.S.A). Plastic dishes and plates including Transwells with 12-mm 0.45-μM cellulose membranes were purchased from Corning (Corning, NY, USA). All culture medium and supplements were purchased from Invitrogen (Carlsbad, CA). Ibuprofen was purchased from Sigma-Aldrich (St. Louis, MO). The selective COX-1 inhibitor, FR12047 (Cat# 236005) and the selective COX-2 inhibitor II (cat# 236012) were purchased from EMD Millipore (Millipore, Billerica, MA). Fluorescein Dextran 3000 MW was purchased from Molecular Probe (ThermoFisher Scientific, Waltham, MA).

### Cell culture and ibuprofen treatment

The UROsta, UM-UC-5 and UM-UC-9 cells were cultured as described [9]. The normal human trophoblast B6Tert-1 cells were cultured on collagen I-coated dishes as described [30]. Human breast cancer JIMT-1 cells were cultured in EMEM medium supplemented with 10 % fetal bovine serum (FBS), sodium pyruvate, non-essential amino acids, and vitamins. All cells were incubated at 37 °C in a humidified atmosphere of 5 % CO_2_ in air.

Ibuprofen was dissolved in dimethyl sulfoxide (DMSO) at a concentration of 0.5 M as a stock. Cells were seeded in or grown to confluence in 12-well plates, and were treated with ibuprofen at different doses or times. For the experiments using the B6Tert-1 cells, confluent cultures were treated with 2 mM ibuprofen for 24 h and were then trypsinized and seeded in 12-mm Transwells for continued growing and transepithelial electrical resistance (TEER) measurement. DMSO was used as a solvent control for the ibuprofen treatment.

### Establishment of cell lines with prostasin expression-silencing or prostasin over-expression

Preparations of B6Tert-1 cells with the human prostasin expression silenced using short interfering RNAs (siRNA) were described previously [30]. Construction of B6Tert-1 cell lines with a stable over-expression of prostasin (B6/Pro) or the vector alone (B6/Vec) was described previously, as well [30]. The expression of prostasin in the B6/Pro cells is under the control of the cytomegalovirus (CMV) promoter and is also regulated by the tet repressor, allowing the induction of prostasin expression upon the addition of tetracycline (1 μg/ml) in the culture medium. JIMT-1 cells stably over-expressing prostasin without the tet repressor (JIMT-1/Pro) or harboring the vector alone (JIMT-1/Vec) were constructed using the method described previously [30]. Transient expression of prostasin in the UROtsa cells was accomplished by using a lentivirus harboring the human prostasin cDNA (pLVX-Pro) in the pLVX-Puro lentiviral vector (Clontech Laboratories, Inc.). A lentivirus with the pLVX-Puro vector alone was used as a control.

### Western blot analysis

The procedures for western blot analysis were described previously [31]. Briefly, after the treatment cells were washed with PBS and lysed in RIPA buffer for 15–30 min at 4 °C with rocking. The supernatants were collected by centrifugation of the cell lysates at 10,000 ×g for 10 min. Protein concentrations were determined using a DC Protein Assay kit (Bio-Rad, Hercules, CA). Equal amounts of total protein for each sample were analyzed on SDS-PAGE and electro-transferred to a nitrocellulose membrane. The membranes were blocked with 5 % non-fat milk in TBS-T (20 mM Tris-HCI, pH 7.4, 137 mM NaCl, 0.1 % Tween-20), and incubated with the appropriate primary antibodies at 4 °C for overnight. On the next day, the membranes were washed with TBS-T and blotted with an appropriate secondary antibody conjugated to the horseradish peroxidase (Promega, Madison, WI) for 1 h at room temperature. The membranes were then washed again before an enhanced-chemiluminescence reaction (ECL, Pierce Biotechnology, Inc., Rockford, IL) and exposed to X-ray films. The primary antibodies used were human prostasin (1:4,000, Ref. 3), tubulin (1:5,000, Sigma-Aldrich), glyceraldehyde 3-phosphate dehydrogenase (GAPDH, 1:5,000, Santa Cruz Biotechnology, Inc., Santa Cruz, CA), matriptase (mouse monoclonal, 1:4,000, Santa Cruz Biotechnology), matriptase (rabbit polyclonal, 1:4,000, Bethyl Laboratories, Inc., Montgomery, TX) and COX-2 (1:1,000, R&D System, Inc., Minneapolis, MN).

### Reverse-transcription and real-time quantitative polymerase chain reaction (RT-qPCR)

Cells at confluence in a 12-well plate were treated with ibuprofen at different doses or DMSO for different time periods. The total RNA was isolated using the TRIzol® reagent (Invitrogen). The procedures for RT-qPCR analysis of prostasin, matriptase, COX-2, GAPDH mRNA expression have been described previously [16, 17]. The relative quantity of each gene’s transcript was calculated using the ΔCt method (Bio-Rad Application Guide) to normalize to the quantity of the GAPDH transcript.

### Transepithelial electrical resistance (TEER) measurement

For the ibuprofen-treated cells: The B6Tert-1 cells were treated with ibuprofen (2 mM) or DMSO for 24 h and then trypsinized and seeded at 2 ×10^5^/insert in 12-mm diameter Transwell inserts. On the next day, the transepithelial electrical resistance (TEER) was measured using an Epithelial Voltohmmeter (EVOM, World Precision Instruments, Inc., Sarasota FL). After the measurement, the ibuprofen-treated cells were treated with either a prostasin antibody, or a pre-immune rabbit serum (as a control), or left alone without any treatment. The TEER was measured every day for 5 days and the medium was changed every 2 days with the addition of the prostasin antibody or the pre-immune rabbit serum.

For cells with transient prostasin-silencing: 24 hours after the transfection of the prostasin siRNA (100 pM) or a random siRNA (100 pM), cells were seeded at 2 ×10^5^/insert in the Transwell inserts as described above. The TEER was measured at 24 h after seeding and every day thereafter for a total of 3 days.

For cells with stable prostasin over-expression: The B6/Vec and B6/Pro cells were seeded at 2 ×10^5^/insert in the Transwell inserts as described above. The TEER was measured every day for 5 days and the medium was changed every two days. Tetracycline was added at seeding to induce the prostasin expression. Tumor necrosis factor alpha (TNFα) was added on day 2 after the measurement of TEER to further enhance the CMV promoter for achieving a higher prostasin expression.

### Paracellular flux (permeability) assay

A fluorescein isothiocyanate (FITC)-labelled dextran (MW 3000) was used and the experiments were performed as described previously with some modifications [32]. The B6Tert-1 cells with silenced prostasin expression and the corresponding control cells were seeded at 2 ×10^5^/insert in 12-mm diameter Transwell inserts. The TEER was monitored every day after the seeding until the TEER reached a plateau. The FITC-dextran was added to the apical side of the monolayer cells at a final concentration of 30 μM, and the culture was incubated for overnight. After the incubation, 100 μl of the medium were collected from the basal side of the monolayer cells and used for measuring the intensity of the fluorescence at wavelengths of Ex494/Em520 nanometers using a Cary Eclipse Fluorescence Spectrophotometer (Varian, Inc., Walnut Creek, CA, USA). A higher intensity of the fluorescence in the basal medium corresponds to a greater amount of the FITC-dextran passing through the paracellular space from the apical side of the cells to the basal side of the cells.

### Statistical analysis

Data are presented as mean ± SD. A statistically significant difference among group means was determined by one-way ANOVA coupled with the TukeyHSD post hoc test. A statistically significant difference was defined as when *p* < 0.05.

## Results

### Regulation of prostasin expression by ibuprofen

**Fig. 1:**
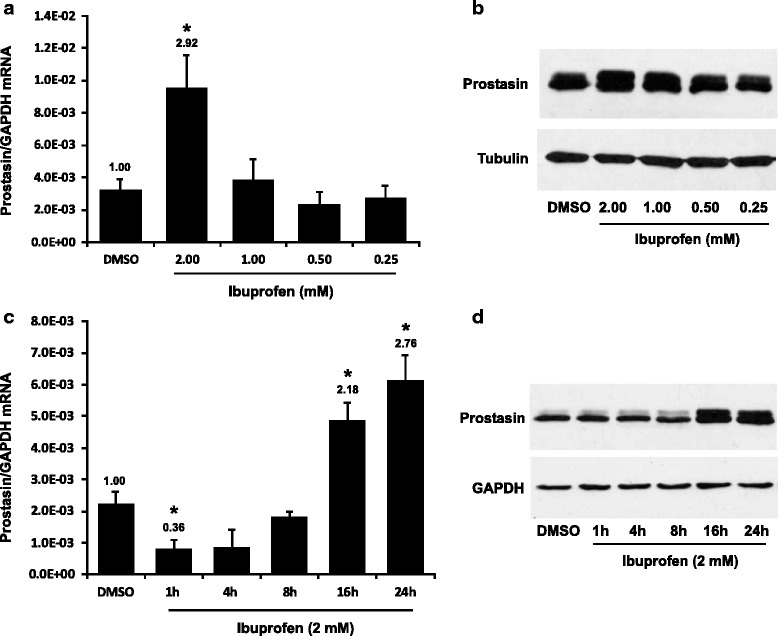
Effects of ibuprofen on prostasin expression. **a** & **b** the UROtsa cells were treated with ibuprofen for 24 h at different dosages as indicated. **a** RT-qPCR (*n* = 3). **b** western blotting (representative image from three experiments). **c** & **d** the UROtsa cells were treated with 2 mM ibuprofen for different time periods as indicated. **c** RT-qPCR (*n* = 3). **d** western blotting (representative image from three experiments). The numbers above each bar (**a**) & (**c**) indicate the fold change of the expression as compared to the DMSO control. One-way ANOVA and Tukey’s post hoc were used for data analysis, and *p* < 0.05 was considered statistically significant. The asterisk denotes *p* <0.05 between IBU treated and DMSO control cells

Previously, we have shown that the prostasin expression is down-regulated in lipopolysaccharide (LPS)-induced bladder inflammation [16]. Here we investigated if ibuprofen (IBU), a non-steroidal anti-inflammatory drug (NSAID), could affect prostasin expression by the inflammation/anti-inflammation pathways. The UROtsa normal human urothelial cells were treated with ibuprofen at different doses ranging from 0.25 mM to 2 mM for 24 h. The expression of prostasin was analyzed at the mRNA level by means of reverse-transcription/quantitative polymerase chain action (RT-qPCR), and at the protein level by means of western blotting using a prostasin-specific antibody. The prostasin expression was up-regulated by IBU at the high dose (2 mM), at both the mRNA and the protein levels (Fig. 1a & b). The up-regulation of prostasin by IBU was not apparent until 16 h after the IBU treatment (Fig. 1c & d). Interestingly, the prostasin mRNA expression was initially down-regulated at 1 h after the IBU treatment, with an up-regulation in the late phase (16–24 h). There however, was not a corresponding reduction of the prostasin protein in the early sampling of the IBU-treated cells. This probably is a reflection of a rather stable half-life of the prostasin protein present in the cells prior to the IBU treatment. It is unclear why there was a two-phased regulation of the prostasin gene expression in response to the IBU treatment.

### Regulation of COX-2 expression by ibuprofen

**Fig. 2:**
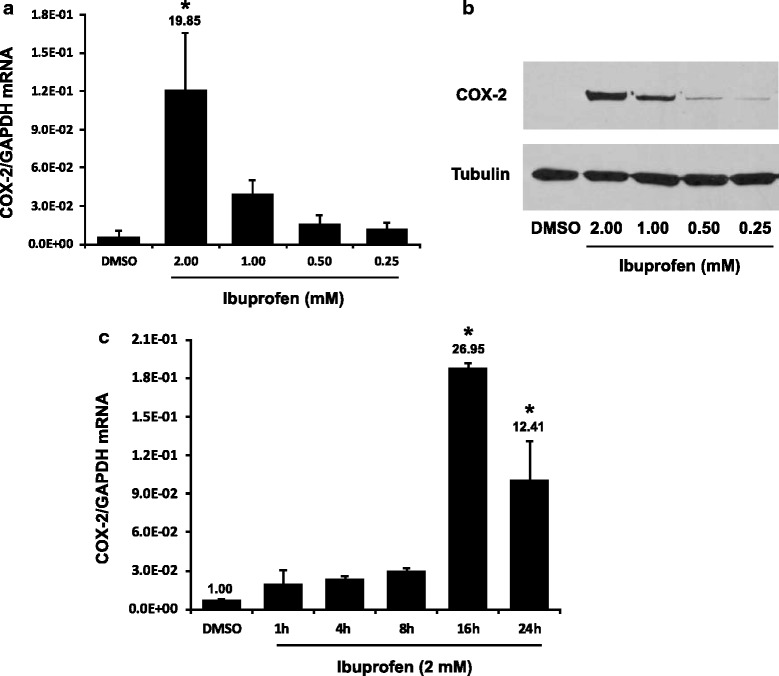
Ibuprofen up-regulates COX-2 expression. **a** & **b** the UROtsa cells were treated with ibuprofen for 24 h at different dosages as indicated. **a** RT-qPCR (*n* = 3). **b** western blotting (representative image from three experiments). **c** RT-qPCR (*n* = 3), the UROtsa cells were treated with 2 mM ibuprofen for different periods of time as indicated. The numbers above each bar (**a**) & (**c**) indicate the fold change of the expression as compared to the DMSO control. One-way ANOVA and Tukey’s post hoc were used for data analysis, and *p* < 0.05 was considered statistically significant. The asterisk denotes *p* < 0.05 between IBU treated and DMSO control cells

The basal expression of COX-2 is moderate at the mRNA level in the UROtsa cells (Fig. 2a, DMSO-treated, equivalent to 0.6 % of the GAPDH level), and not detectable at the protein level (Fig. 2b, DMSO-treated). The COX-2 expression was greatly induced by IBU in the UROtsa cells (Fig. 2a), up to 12.1 % of the GAPDH level or at 19.85 fold over the control. The IBU induction of COX-2 expression was dose-dependent at both the mRNA and the protein levels. Furthermore, the COX-2 expression was induced in a time-dependent manner with the highest increase at 16 h after the IBU treatment (Fig. 2c). The coincidental time course of prostasin and COX-2 induction by the IBU treatment may suggest a causal relationship of the two induced genes. On the other hand, in a human breast cancer cell line JIMT-1, prostasin expression was up-regulated by 1 mM ibuprofen at 24 h of treatment, but without a significant induction of COX-2 expression (data not shown), suggesting that the up-regulation of prostasin expression by IBU does not require a concomitant COX-2 up-regulation. We also evaluated the COX-1 expression in the UROtsa cells. The basal expression level of COX-1 is minimal at less than 0.01 % of the GAPDH level; and the COX-1 expression was also increased in response to the IBU treatment, but only marginally to no more than 0.03 % of the GAPDH level (data not shown).

### Effect of selective COX inhibitors on prostasin expression

**Fig. 3:**
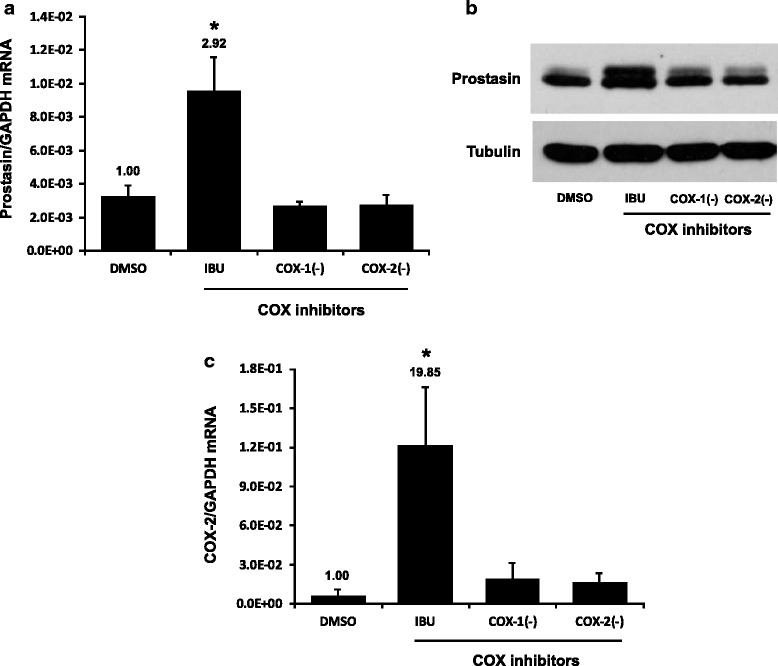
Selective COX inhibitors did not affect prostasin expression. The UROtsa cells were treated with COX inhibitors for 24 h. IBU: ibuprofen, 2 mM; COX-1(−): COX-1 inhibitor, 10 μM; COX-2(−): COX-2 inhibitor, 10 μM. **a** & **c** RT-qPCR (*n* = 3). The numbers above each bar indicate the fold change of the expression as compared to the DMSO control. Note: the bars of DMSO and IBU in (**a**) and (**c**) are the same of those in Fig. 1a and Fig. 2a, respectively. One-way ANOVA and Tukey’s post hoc were used for data analysis, and *p* < 0.05 was considered statistically significant. The asterisk denotes *p* < 0.05 between IBU treated and DMSO control cells. **b** western blotting (representative image from three experiments)

Ibuprofen is a non-selective inhibitor of both the COX-1 and COX-2 enzymes. We sought to determine if ibuprofen’s action on prostasin expression was mediated by inhibiting either the COX-1 or COX-2 enzyme activity. The selective COX-1 inhibitor, FR12047 has an IC50 of 28 nM and a ~2300 fold selectivity towards the COX-1 enzyme over the COX-2 enzyme. The selective COX-2 inhibitor II has an IC50 of 4 nM and a ~28,500 fold selectivity towards the COX-2 enzyme over the COX-1 enzyme. Both selective COX inhibitors were used for treating the UROtsa cells as indicated in Fig. 3. Neither inhibitor up-regulated prostasin or COX-2 expression.

### Effect of prostasin on the transepithelial electrical resistance

**Fig. 4:**
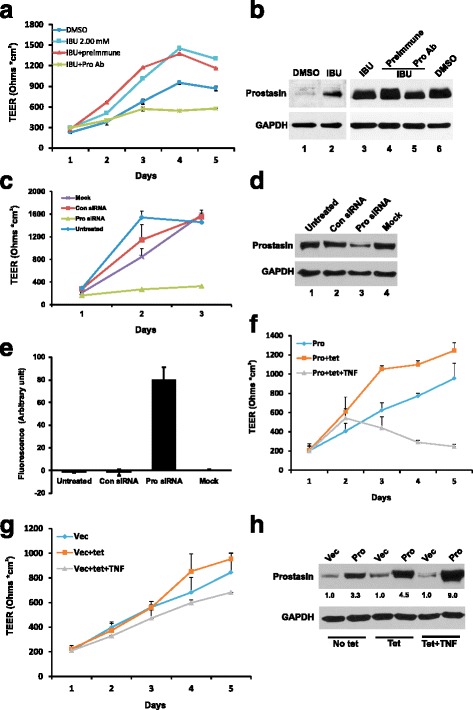
Transepithelial electric resistant (TEER) measurement and permeability assay. The B6Tert-1 cells were first treated with 2 mM ibuprofen for 24 h and seeded in Transwells to develop cell-cell contact and tight junctions. The TEER was measured using an EVOM device as described in the Methods. **a** TEER of B6Tert-1 cells treated with DMSO (used as a solvent control), or ibuprofen, or a prostasin antibody (Pro Ab), or a pre-immune rabbit serum (used as a control). ANOVA: *p* < 0.05. **b** Western blot analysis of prostasin expression in B6Tert-1 cells treated with 2 mM ibuprofen for 24 h (Lanes 1–2); or grown in Transwells for 5 days (Lanes 3–6). **c** TEER of B6Tert-1 cells treated with a prostasin-specific siRNA. Untreated: cells were not treated with any reagent; Con siRNA: a random siRNA used as a control; Pro siRNA: the prostasin-specific siRNA; Mock: cells were treated with the transfection reagent Lipofectamine 2000 only. ANOVA: *p* < 0.05. **d** Western blot analysis of prostasin expression in B6Tert-1 cells after silencing prostasin expression. **e** Permeability of B6Tert-1 cells to FITC-dextran (*n* = 3). The prostasin siRNA-treated cells had the most FITC-dextran in the basal medium of the cells. ANOVA: *p* < 0.05. **f** TEER of B6Tert-1 cells expressing different amounts of prostasin. Pro: prostasin; tet: tetracycline; TNF: tumor necrosis factor (alpha). ANOVA: *p* < 0.05. **g** TEER of B6Tert-1 cells harboring the vector alone (Vec). ANOVA: *p* > 0.05. **h** Western blot analysis of prostasin expression in B6Tert-1 under tet or tet + TNF-α treatment. The numbers under each lane indicate the fold change of the expression as compared to the vector control (Vec)

Prostasin is required for maintaining the epithelial barrier function and for epidermal terminal differentiation [20, 23]. We hypothesized that the ibuprofen-induced prostasin could help maintain the epithelial barrier integrity by promoting tight junction formation and maintenance. Such a functional output can be manifested by an increased transepithelial electrical resistance (TEER). To evaluate IBU’s effects on the TEER via prostasin up-regulation, we chose to use a normal human trophoblast cell line B6Tert-1 because the B6Tert-1 cells establish tight junctions over the course of monolayer expansion with a sharper increase of the TEER giving the assay an increased and requisite robustness. The UROtsa cells, however, were not able to form tight junctions with an increase in TEER over the cell culturing period. As shown in Fig. 4a, compared with the B6Tert-1 cells treated with DMSO, the B6Tert-1 cells treated once with 2 mM ibuprofen for 24 h established a higher TEER and this effect lasted for at least 5 days. An increased expression of prostasin in the B6Tert-1 cells following the IBU treatment was confirmed by western blotting (Fig. 4b, lanes 1–2). The addition of a functionally blocking prostasin antibody to the ibuprofen-treated cells however, prevented the TEER increase and counteracted IBU’s effect (Fig. 4a). The addition of a control pre-immune rabbit serum did not affect IBU’s effect on the TEER. The expression of prostasin at the end of the TEER measurements (day 5) was analyzed by western blotting (Fig. 4b, lanes 3–6). The cellular prostasin protein level was reduced due to the addition of the prostasin antibody in the culturing medium. The reduced prostasin protein level was accompanied with a lower TEER as indicated in Fig. 4a. Transient silencing of the prostasin gene expression by siRNA in the B6Tert-1 cells also impaired on the TEER gain (Fig. 4c), underscoring the role of prostasin in maintaining the integrity of the epithelial tight junction. Reduction of the prostasin protein expression in the B6Tert-1 cells treated with the prostasin-specific siRNA was confirmed by western blotting (Fig. 4d). The change of the TEER as a result of prostasin expression silencing in the B6Tert-1 cells was accompanied with a change of permeability to macromolecules. The prostasin-reduced B6Tert-1 cells allowed the FITC-labeled dextran to pass through the paracellular space from the apical side to the basal side of the monolayer cells, as indicated by the higher intensity of fluorescence in the basal medium of these cells whereas the control cells essentially did not allow the FITC-dextran to pass through (Fig. 4e). This observation indicates that the TEER change along with the prostasin expression silencing was the result of a tight junction compromise.

Previous studies showed that either prostasin knockout or over-expression in the mouse skin could impair the skin epidermal barrier function [23, 33]. In order to better understand prostasin’s function at the mechanistic level in tight junction regulation, we studied a stable B6Tert-1 cell line (B6/Pro) that expresses prostasin under the CMV (cytomegalovirus) promoter with tet-on regulation [30]. Since the CMV promoter activity can be enhanced by tumor necrosis factor alpha (TNF-α, [34]), the cells were also treated with TNF-α in some experiments. When the prostasin expression was moderately induced in the B6/Pro cells with tetracycline (tet, 1 μg/ml), the cells presented a higher TEER than those without the tetracycline treatment (Fig. 4f). However, when the prostasin expression was further induced with both tetracycline and TNF-α (5 ng/ml), the B6/Pro cells had an acute reduction in the TEER; indicating a compromised tight junction integrity. The TEER was not affected in the B6/Vec control cells under similar treatments, i.e., tetracycline induction alone or in combination with TNF-α (Fig. 4g). Treating the B6/Pro and B6/Vec cells with TNF-α alone also had no effect on the TEER (data not shown). The amount of prostasin protein in the B6/Vec cells and the B6/Pro cells under tetracycline treatment without or with the addition of TNF-α is shown in Fig. 4h. The TEER changes observed in the B6/Pro cells can therefore, be attributed to the prostasin over-expression changes. These results indicate that a regulated amount of prostasin expressed in the cells is critical for the establishment and maintenance of the tight junction; whereas too little or too much prostasin expression is detrimental to the epithelial tight junction integrity.

### Regulatory interactions between prostasin and matriptase serine proteases

**Fig. 5:**
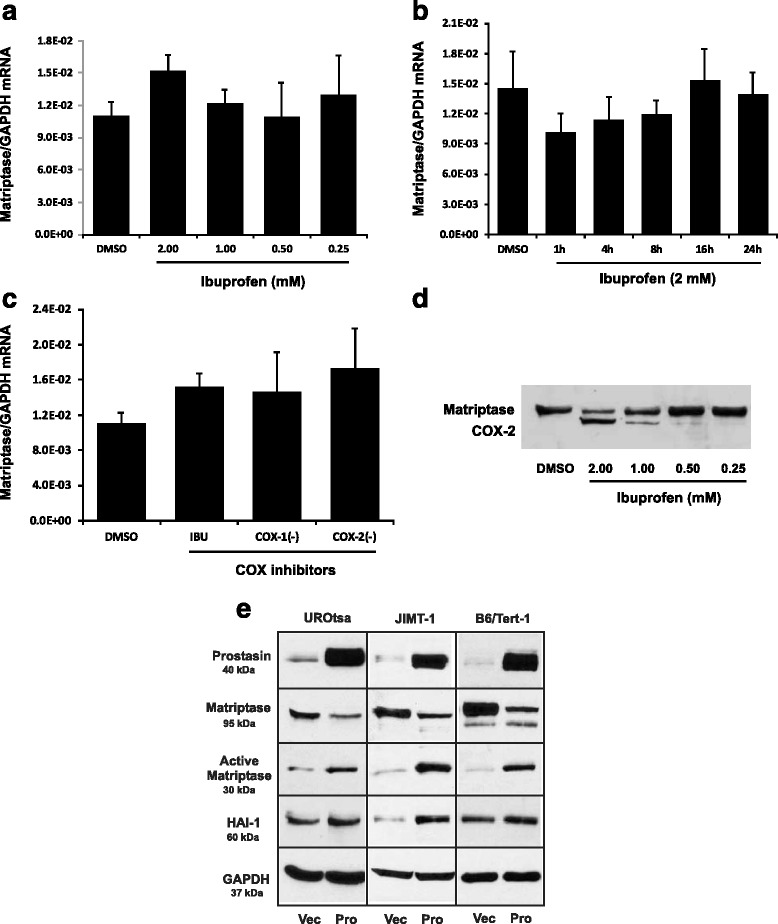
Effect of COX inhibitors and prostasin on matriptase expression. **a** & **b** RT-qPCR (*n* = 3). The UROtsa cells were treated with different dosages of ibuprofen for 24 h or at 2 mM ibuprofen for different time periods. **c** RT-qPCR (*n* = 3). The UROtsa cells were treated with COX inhibitors for 24 h. IBU: ibuprofen, 2 mM; COX-1(−): COX-1 inhibitor, 10 μM; COX-2(−): COX-2 inhibitor, 10 μM. **a**, **b**, and **c** The numbers above each bar indicate the fold change of the expression as compared to the DMSO control. ANOVA: *p* > 0.05. Note: the bars of DMSO and IBU in (**c**) are the same of those in (**a**). **d** Western blot analysis of matriptase in UROtsa treated with different dosages of ibuprofen for 24 h as indicated. Note the samples on the membrane were blotted with the anti-matriptase antibody after blotting with anti-COX-2 and anti-tubulin antibodies shown in Fig. 2b. The previously blotted COX-2 protein bands is shown along with the matriptase protein bands. **e** Western blot analysis of prostasin, matriptase, HAI-1 expression in stable or transient prostasin over-expressing cell lines as indicated. Vec: vector control; Pro: prostasin over-expressing lines

As prostasin and matriptase regulate each other’s functions reciprocally, we sought to investigate if an ibuprofen treatment would bring forth accompanying changes in matriptase expression or function along with the prostasin expression and function changes. As shown in Fig. 5 in the UROtsa cells, the matriptase expression was mostly regulated at the protein level rather than at the mRNA level by ibuprofen or selective COX inhibitors. The matriptase mRNA level was not changed (Fig. 5a–c), but the 95-kDa matriptase zymogen was down-regulated with increasing doses of ibuprofen in the treatment (Fig. 5d). It is not clear if ibuprofen directly affected the matriptase zymogen or indirectly via the increased prostasin protein expression.

To determine if the increased prostasin can cause a down-regulation of the matriptase zymogen, we transiently infected UROtsa cells with a prostasin-expressing lentivirus (pLVX-Pro). The matriptase zymogen is a 95-kDa type-II membrane protein on epithelial cell membranes. Upon cleavage/activation, the C-terminal serine protease domain could still be non-covalently linked with the “stem” part of the matriptase protein and/or the inhibitor HAI-1 on the cell membrane; without being released into the culturing medium [35]. As shown in Fig. 5e, the left panel; prostasin over-expression caused a quantity reduction of the 95-kDa matriptase zymogen in the UROtsa cells, but a quantity increase of a 30-kDa matriptase fragment recognized by the antibody capable of recognizing the C-terminal serine protease domain of matriptase. To determine if this phenotype exists in other cell lines, we stably over-expressed prostasin in the human breast cancer cell line JIMT-1 and the B6Tert-1 trophoblast cells. Similar results were obtained as shown in Fig. 5e, the middle and right panels, that upon prostasin over-expression, the 95-kDa matriptase zymogen was reduced in quantity but the 30-kDa matriptase protease domain was increased in quantity. These results indicated that prostasin may be responsible for the cleavage of the 30-kDa matriptase serine protease domain either directly or indirectly via another intermediate protease; and the IBU-induced prostasin could, at least in part, be responsible for the reduced matriptase zymogen expression shown in Fig. 5d.

We also evaluated the expression level of the hepatocyte growth factor activator inhibitor type I (HAI-1) in these cells that over-expressed prostasin, either transiently or stably. HAI-1 is expressed as a membrane-associated Kunitz-type protein which binds to and inhibits the activity of many serine proteases including prostasin and matriptase. In the prostasin over-expressing UROtsa, JIMT-1 and B6Tert-1 cells, the quantity of the membrane-associated HAI-1 protein was increased, possibly as a result of an increased prostasin expression on the cell membrane (Fig. 5e). The increased prostasin could bind more HAI-1 resulting in the retention of HAI-1 on the cell membrane, rather than being secreted into the culturing medium [36]. The mRNA level of HAI-1 was not significantly affected in these cells (data not shown).

### Regulation of prostasin expression by IBU in bladder cancer cell lines

**Fig. 6:**
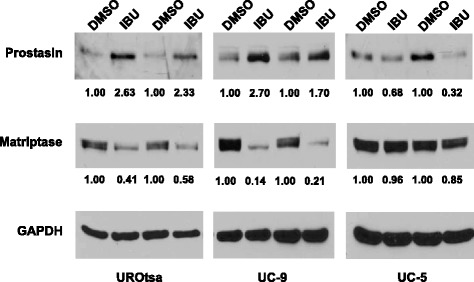
Western blot analysis of prostasin and matriptase expression in UROtsa and human bladder transitional cell carcinoma cell lines (UC-9 and UC-5). The UROtsa cells were treated with 2 mM ibuprofen and cancer cells were treated with 0.5 mM ibuprofen for 24 h. Twenty-five micrograms of total protein from each sample were loaded in each lane. Samples from two separated experiments were shown. The numbers under each lane indicated the fold change of the expression as compared to the DMSO control. IBU: ibuprofen. GAPDH was used as a loading control

We previously reported that the prostasin expression was down-regulated in high-grade bladder cancers. A loss of prostasin expression in bladder cancer cell lines is associated with epithelial-mesenchymal transition [9]. Here, we examined if IBU can up-regulate prostasin expression in bladder cancer cell lines. Similar to what was seen in the UROtsa cells, an up-regulation of the prostasin protein expression and a down-regulation of the matriptase protein expression in the UM-UC-9 human bladder cancer cells were observed after an IBU treatment, as shown in Fig. 6. Rather different changes, however, were observed in the UM-UC-5 human bladder cancer cells. The prostasin protein expression was decreased after the IBU treatment in UM-UC-5, without accompanying changes of the matriptase expression (Fig. 6). The results indicated that bladder cancer cells may respond to ibuprofen treatment differently depending on whether the specific cancer cells have retained the proper pathways for the responses seen in the normal epithelial cells.

## Discussion

We reported here that ibuprofen, a non-steroidal anti-inflammatory drug and a non-selective inhibitor of cyclooxygenases, up-regulated the prostasin expression in epithelial cells; and in turn increased the transepithelial electrical resistance (TEER) of the epithelial cells. In vitro, the TEER change reflects a change in the integrity of epithelial cell tight junctions since the TEER is primarily determined by the apical tight junctions. The higher the TEER the stronger the tight junctions there are between epithelial cells. A loss of the barrier integrity in the epithelium may promote fluid movement from the interstitial regions into the lumen spaces, or facilitate the movement of toxic and carcinogenic chemicals from the lumen through the paracellular passage into the underlying tissue and blood stream causing further damages. Therefore, maintaining the intact epithelial integrity is essential for a healthy body against potentially toxic environmental insults.

The serine protease prostasin plays important roles in maintaining the integrity of the epithelium via regulating the tight junctions at the cell-cell contacts [20, 23], in taming innate epithelial inflammation via attenuating the expression of inducible nitric oxide synthase (iNOS) in vivo, and in the regulation of the iNOS and COX-2 gene expression in vitro [16–18]. If epithelial cells can maintain a sufficient prostasin expression under stress such as an inflammation to help maintain the epithelium integrity, the damage to the epithelium and the underlying tissue may be reduced or minimized. Knocking out the prostasin gene expression in the mouse skin compromised epithelial cell tight junctions leading to a life-threatening dehydration of the neonatal skin [23]. On the other hand, over-expression of prostasin in epithelial cells to or beyond a certain level decreases the TEER (Fig. 4); which ironically is a phenotype also associated with a prostasin down-regulation. In our results, either a reduced or an over-abundant prostasin expression is detrimental to the epithelium structures and functions (Fig. 4). It is vital that the prostasin expression level and its functions are tightly controlled in epithelial cells.

Matriptase intimately interacts with prostasin in the functional roles at the epithelium and the two proteases reciprocally activate each other from their respective zymogens [19, 20, 32]. The matriptase expression was unaffected by ibuprofen at the mRNA level but its zymogen was down-regulated by ibuprofen. In our model cell lines, over-expression of a recombinant prostasin alone was capable of inducing matriptase activation and zymogen down-regulation (Fig. 5e) so the ibuprofen-induced prostasin may very well be responsible for the matriptase activation and zymogen down-regulation in the ibuprofen-treated cells. While the increased prostasin can activate matriptase, it is also possible that the cognate serine protease inhibitor(s) for both proteases, e.g., HAI-1, is competed off matriptase by the increased prostasin. In other words, the dynamic of the protease-inhibitor network, prostasin ↔ HAI-1 ↔ matriptase, is tipped in favor of matriptase to be more active. A reduced HAI-1 availability to inhibit matriptase will enhance matriptase auto-activation and the activated matriptase will activate more prostasin. Our current results are consistent with that of a previous study [32] in which either the co-expression of prostasin and matriptase in the presence of the inhibitor HAI-1 or the addition of a recombinant prostasin to the Caco-2 cells could generate the 30-kD active matriptase protease domain. A keenly balanced regulation of the two serine proteases, prostasin and matriptase, and their inhibitor, HAI-1 would therefore be important for maintaining intact epithelial tight junctions.

Prostasin expression was down-regulated by ibuprofen at 1 h after the treatment in the UROtsa cells. A recent report by Lichtenberger et al. [37] revealed that ibuprofen could interact with phosphatidylcholine to transiently change cell membrane physical properties leading to increased cell permeability. Such a mechanism of action would lead to a condition that mimics a transient inflammation-like state in the UROtsa cells. Consequently, prostasin expression was reduced as a response to this “inflammation mimicry”. Alternatively, the ibuprofen-phospholipid interaction could affect cell signaling pathways that lead to the down-regulation of the prostasin expression in the early phase of the ibuprofen treatment. Whether an early down-regulation of prostasin by ibuprofen is mediated by such pathways requires further investigation.

In vitro, the level of prostasin expression changes with the cell density, i.e. a lesser prostasin expression in sparsely-cultured cells and a higher prostasin expression in densely-cultured cells (Chen, unpublished results). In vivo, a proper prostasin expression is indispensable for the placenta labyrinth maturation, during the differentiation of cytotrophoblasts to syncytiotrophoblasts [38–40]. Furthermore, prostasin expression is often lost in poorly differentiated cancers. These results suggested that prostasin is expressed less in proliferating cells but expressed more in differentiating cells. Treating epithelial cells with the anti-inflammatory drug ibuprofen may trigger the cell repair/differentiation signaling pathways, resulting in prostasin expression up-regulation. The response of epithelial cells to an ibuprofen treatment varies depending on the cell type and the cell states, i.e., in a proliferating state or a repair/differentiation state. It would follow simply from the view point of prostasin expression regulation by ibuprofen that only certain cancer patients with certain types of cancer would benefit from taking ibuprofen as an adjuvant treatment.

Ibuprofen at a high dose up-regulates prostasin expression and in turn increases the TEER. Even though there was a significant increase of COX-2 expression along with the up-regulation of prostasin, it is unlikely that the increased COX-2 expression caused the increase of prostasin expression since in the JIMT-1 cells there was an increased prostasin expression by ibuprofen without an accompanying induction of COX-2 (data not shown). At least, the COX-2 induction is not a requisite for the prostasin induction by ibuprofen and the two ibuprofen-induced molecular events do not seem to be coupled.

Ibuprofen is used to reduce inflammation and pain based on an accepted mechanism of inhibiting cyclooxygenases. But other indications have been proposed for ibuprofen, such as its use as an anti-cancer agent. The molecular mechanisms for such indications may be far less defined and may have very little to do with COX inhibition. A recent study by Sun et al. [41] suggested that phosphor-ibuprofen (P-I, MDC-917), a derivative of ibuprofen, has an anti-cancer function via the regulation of oxidative stress and redox homeostasis involving the Trx system. Breast cancer cells treated with P-I had an increased level of oxidized Trx-1 and a reduced thioredoxin reductase (TrxR) activity. The downstream changes were a suppressed NF-κB activity and activated p38 and JNK kinase cascades. The P-I’s anti-cancer activity is Trx-1-dependent since Trx-1 knockdown diminished the P-I’s anti-cancer activity. Prostasin has not been studied in the context of oxidative stress nor identified as a player in the Trx system. Prostasin, however, is an inflammatory “negative” protein and down-regulated during inflammation, but up-regulated by the anti-inflammatory drug ibuprofen. Prostasin has been suggested to inhibit tumor invasion and metastasis, and prevent epithelial-mesenchymal transition. Epidemiological studies have shown that ibuprofen reduces the progression and recurrence of various types of cancers [28]. It is reasonable to propose that up-regulating prostasin expression by ibuprofen may be a mechanism of ibuprofen in its anti-cancer action.

Previously, we have shown that prostasin promoter methylation was a mechanism for the reduced expression of prostasin in bladder cancer cells. The prostasin gene promoter is minimally heterogeneously methylated in the UM-UC-9 and unmethylated in the UM-UC-5 at a site critical for prostasin expression and both cell lines express a high level of prostasin [9]. The prostasin promoter in the UM-UC-9 appeared to be significantly more active than that in the UM-UC-5 when the prostasin mRNA transcripts were evaluated in these cell lines [9], suggesting that mechanisms other than promoter methylation be at play for the prostasin transcriptional activity. The more active prostasin promoter in the UM-UC-9 is thus responding to the ibuprofen treatment in the current study with the observed up-regulation. De-methylation and/or histone deacetylase (HDAC) inhibition can reactivate the prostasin promoter as we have shown for various cancer cells [7, 9, 11]. De-methylation and/or HDAC inhibition agents may be required prior to ibuprofen intervention to reactivate and up-regulate prostasin in bladder cancer cells presenting hypermethylation of the prostasin promoter.

## Conclusion

Prostasin expression must be tightly regulated in the epithelium to maintain a proper barrier function of the tissue. The NSAID Ibuprofen regulates prostasin expression and function in epithelial cells, and reduces the progression and recurrence of certain cancers. Our results bring the epithelial extracellular membraneserine proteases prostasin and matriptase into the potential molecular mechanisms of the anticancer effect of NSAIDs.

## Abbreviations

ANOVA: Analysis of variance
B6Tert-1: Normal human trophoblasts
CMV: Cytomegalovirus
COX: Cyclooxygenase
DMSO: Dimethyl sulfoxide
DNA: Deoxyribonucleic acid
EMEM: Eagle’s minimal essential medium
EMT: Epithelial-mesenchymal transition
FBS: Fetal bovine serum
FITC: Fluorescein isothiocyanate
GAPDH: Glyceraldehyde 3-phosphate dehydrogenase
GPI: Glycosylphosphatidylinositol
HAI-1: Hepatocyte growth factor activator inhibitor type 1
HAI-2: Hepatocyte growth factor activator inhibitor type 2
HDAC: Histone deacetylase
hTert: Human telomerase reverse transcriptase
IBU: Ibuprofen
iNOS: Inducible nitric oxide synthase
JIMT-1: Herceptin-resistant breast cancer cells
LPS: Lipopolysaccharide
NGF: Nerve growth factor
NSAID: Nonsteroidal anti-inflammatory drug
PN-1: Protease Nexin-1
RIPA: Radioimmunoprecipitation assay buffer
RNA: Ribonucleic acid
RT-PCR: Reverse transcription polymerase chain reaction
SDS-PAGE: Sodium dodecyl sulfate polyacrylamide gel electrophoresis
siRNA: Small interfering RNA
SREBP: Sterol regulatory element-binding proteins
TBS-T: Tris-buffered saline-tween 20
TEER: Transepithelial Electrical Resistance
Tet: Tetracycline
TGF-β1: Transforming growth factor beta 1
TNF-α: Tumor necrosis factor alpha
Trx: Thioredoxin
TrxR: Thioredoxin reductases
UM-UC-3: Human bladder transitional cell carcinoma
UM-UC-9: Human bladder transitional cell carcinoma
UROtsa: Normal human urothelium cells
